# Maximum Phonation Time in People with Obesity Not Submitted or Submitted to Bariatric Surgery

**DOI:** 10.1155/2019/5903621

**Published:** 2019-12-25

**Authors:** Ana Luara Ferreura Fonseca, Wilson Salgado, Roberto Oliveira Dantas

**Affiliations:** ^1^Department of Medicine, Ribeirão Preto Medical School, University of São Paulo, Av. Bandeirantes 3900 Ribeirão Preto SP, São Paulo 14049-900, Brazil; ^2^Department of Surgery and Anatomy, Ribeirão Preto Medical School, University of São Paulo, Av. Bandeirantes 3900 Ribeirão Preto SP, São Paulo 14049-900, Brazil

## Abstract

**Background:**

Our aim in this investigation was to evaluate maximum phonation time in people with obesity not submitted to surgery and in people with obesity submitted to bariatric surgery and compare it with maximum phonation time of healthy volunteers. The hypothesis was that the reduced maximum phonation time in people with obesity would be corrected after surgery due to weight loss.

**Method:**

Maximum phonation time was evaluated in 52 class III patients (Group A), 62 class III patients who were treated by surgery 3 to 115 months before (Group B), 20 controls (Group C), and 15 class III patients whose maximum phonation time was evaluated before and two to six months after surgery (Group D). Maximum phonation time was measured in the sitting position with the vowels /A/, /I/, and /U/.

**Results:**

Maximal phonation time was shorter in groups A and B compared with that of controls. There was an increase in maximal phonation time after surgery (Group B); however, the difference was not significant when compared with that in group A. In group D, maximal phonation time for /A/ increased after the surgery. In group A, there was a negative correlation between maximal phonation time and weight or body mass index and a positive correlation between maximal phonation time and height. In group B, there was an almost significant positive relation between percentage of weight loss and maximal phonation time for /A/ (*p*=0.08) and /I/ (*p*=0.07). Mean values of spirometry testing (FEV_1_, FVC, and FEV_1_/FVC) in people with obesity (groups A and B), expressed as percentage of the predicted value, were within the normal range.

**Conclusion:**

Compared with healthy controls, maximal phonation time is shorter in people with obesity, with a tendency to increase after bariatric surgery, as a possible consequence of weight loss.

## 1. Introduction

Obesity is an important health problem with increasing prevalence around the world [1, 2]. It is associated with complications and high cost for its prevention and treatment [2].

Pulmonary function is affected by obesity, with lower than expected ventilatory pressures during inspiration and expiration, caused by a reduction in respiratory muscle strength, in functional capacity, in functional residual capacity, in expiratory reserve volume, in vital capacity, in total pulmonary capacity and in lung compliance and volume, as well as by an increase in airway resistance and pulmonary diffusion, heterogeneity of ventilation distribution, and hypercapnic respiratory failure [3–7]. People with obesity have found to have narrowing of the aerodigestive tract, including the hypopharynx, oropharynx, nasopharynx, and nasal cavities, caused by thickening of the lateral pharyngeal walls, medialized tonsillar pillars, and elongated and flaccid velum [8, 9], which may also affect the vocal quality [10–12].

The maximum phonation time (MPT) is an effective tool for the evaluation, diagnosis, and follow-up of patients in treatment for voice alteration [13, 14], which has been reported short in people with obesity [10–12]. There is a controversy about what happens to the MPT after surgical treatment of obesity and consequent weight loss. While some studies have shown increased MPT after surgery [11, 15], others have shown no alteration [16, 17]. Our hypothesis was that (a) obesity decreases MPT, which is improved after surgical treatment of obesity; (b) there is a correlation of the duration of MPT with weight, body mass index (BMI), and neck circumference; and (c) obesity causes abnormalities in forced expiratory volume in one second (FEV_1_) and forced vital capacity (FVC). The objectives of this investigation were to (1) evaluate the MPT in a group of people with obesity and in a group of people with obesity treated by surgery and compare it with a control group of nonobese healthy volunteers; (2) evaluate a group of people with obesity before and after surgical treatment of obesity; and (3) evaluate the correlation of MPT with weight, height, BMI, neck circumference, FEV_1_, FVC, and the relation FEV_1_/FVC (Tiffeneau index) in patients who were not treated surgically.

## 2. Materials and Methods

### 2.1. Participants

The MPT was evaluated in 52 patients with obesity (19 men) aged from 18 to 63 years (42.6 ± 11.6 years) with no surgical treatment of obesity and a BMI greater than 40 kg/m^2^ (group A) and 62 patients with obesity (8 men) aged from 22 to 64 years (41.6 ± 11.0 years) who had been submitted to surgical treatment of obesity. All had a BMI greater than 40 kg/m^2^ before the operation and were evaluated for MPT 3 to 115 months (median: 18 months) after surgery (group B) and 20 healthy controls (3 men), aged 18 to 62 years (40.2 ± 11.9 years), had a BMI between 21.9 and 26.0 kg/m^2^. Weight loss in group B ranged from 11.3 kg to 78.0 kg, with a percentage of weight loss from 9.9% to 50.1% and a mean of 29.1 ± 10.0%. The mean and median age, weight, height, BMI, neck circumference, FEV_1,_ FVC, and FEV_1_/FVC of groups A and B and the mean and median age, weight, height, BMI, and neck circumference of group C are presented in Table 1. In another group of 15 patients with obesity (group D), in 3 men, aged from 21 to 63 years (44.4 ± 12.8 years), MPT was evaluated before the surgery and two to six months (median two months) after the surgery. All individuals in group D had a BMI greater than 40 kg/m^2^ (46.0 ± 5.5 kg/m^2^) before the surgery and a mean BMI of 38.4 ± 4.4 kg/m^2^ after the surgery. No volunteers had respiratory or digestive disease.

Neck circumference was measured with individuals in the sitting position on a chair, with the head in a neutral position, looking straight ahead. A measuring tape was passed around the neck at the level of the thyroid cartilage.

The project was approved by the Human Research Committee of the General Hospital of Ribeirão Preto Medical School, University of São Paulo, Brazil, IRB number HCRP 6741/2014. All volunteers and patients gave written informed consent to participate in the investigation, and the anonymity of the participants was ensured. The principles outlined in the Declaration of Helsinki were followed. The participants did not receive a stipend.

### 2.2. Surgery

Surgical treatment was performed by laparoscopic Roux-en-Y gastric bypass (RYGB). The procedure which has been standardized in our service is performed without a ring, and the length of the biliopancreatic and alimentary loops is of 100 cm each.

### 2.3. Spirometry

Patients of groups A and B were submitted to spirometry. The test was performed on a Pulmonet III spirometer (Sensormedics, Anaheim, CA, USA), according to the recommendations of the American Thoracic Society. The parameters analyzed were FEV_1_, FVC, and the Tiffeneau index (FEV_1_/FVC). The results were expressed according to the percentage predicted for age, height, and sex based on the equations of Crapo et al. [18].

### 2.4. Maximum Phonation Time

MPT was measured in all subjects in the sitting position, with the head in a resting position, looking straight. They were instructed to say the vowels /A/, /I/, and /U/, followed by /S/ and /Z/ after inspiration, in a prolonged and in habitual frequency and intensity. Each measurement was done in triplicate, and the mean of the three measurements of each individual was used for analysis. The MPT was measured in seconds with a digital stopwatch (Technos G183, Brazil).

### 2.5. Statistical Analysis

Statistical analysis was done with the correlation coefficient of Spearman (rho), analysis of covariance (ANCOVA), linear regression, and analysis of multiple effects. They were adjusted for gender and BMI. All analyses were performed with the software SAS® 9.2, and differences were considered significant when *p* ≤ 0.05. The results are presented as mean, median, standard deviation, percentage and, in figure, interquartile range.

## 3. Results

**Figure 1:**
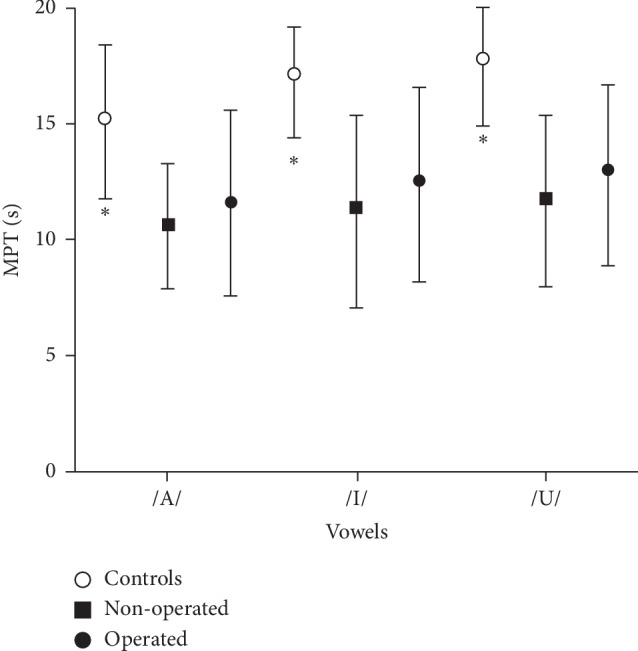
Maximum phonation time (MPT), in seconds, for the vowels /A/, /I/, and /U/, in nonoperated people with obesity (Group A, *n* = 52), in operated people with obesity (Group B *n* = 62), and healthy controls (Group C, *n* = 20). ^*∗*^*p* < 0.05 compared with groups A and B. Results are expressed as mean and interquartile range.

The MPT for the sounds /A/, /I/, and /U/ were longer in the control subjects than in people with obesity (*p* < 0.04), with or without surgery (Table 2). Men and women have the same results with the comparison between groups A and B and group C. The S/Z index was similar in the three groups, A, B, and C (*p* > 0.05). MPT has the tendency to be lower in group A than in group B, but the statistical analysis did not show difference (*p* > 0.40, Figure 1).

In group D, there was a decrease in weight, BMI, and neck circumference after surgery compared with preoperative values. Preoperative MPT was shorter than that in controls, and the MPT for /A/ increased from 11.8 ± 4.5 seconds before the surgery to 13.0 ± 4.8 seconds after the surgery (*p*=0.04, Table 3). There was no difference in the comparison before and after the surgery in MPT for /I/, /U/, or in the S/Z index (*p*=0.30).

In group A, there was a significant negative correlation of MPT with both weight and BMI, and a significant positive correlation between MPT and height (Table 4). Although no correlation was found of the MPT for /A/ and /I/ with neck circumference, there was a negative correlation of the MPT of /U/ with neck circumference (*p*=0.04). No correlation was observed between MPT and FEV_1_, FVC, or FEV_1_/FVC in group A. There was no relation between percentage of weight loss (*p*=0.08 for /A/, *p*=0.07 for /I/, *p*=0.42 for /U/) and time after the surgery (*p*=0.67 for /A/, *p*=0.97 for /I/, *p*=0.65 for /U/) with the MPT.

Mean values of FEV_1_, FVC, and FEV_1_/FVC in people with obesity (groups A and B), expressed as percentage of the predicted value, were considered within the normal range.

## 4. Discussion

As previously demonstrated [10–12], MPT was shorter in obese patients than that in nonobese subjects. Our results showed that, after surgical treatment of obesity and weight loss, there was no significant improvement in the MPT. However, our findings also suggested a small increase in MPT after the surgery, possibly requiring a longer postoperative period or even greater weight loss for the MPT to improve significantly. A previous study showed that it was possible to see improvements in MPT eight months after the surgery [15]. In our study, patients of group D showed an increase in the MPT of the vowel /A/ after the surgery and in group B an almost significant positive relation between percentage of weight loss and MPT for /A/ and /I/.

People with obesity have an altered voice quality, which is perceived as more strangled, hoarse, and breathy compared with nonobese ones [16]. Another study with class III subjects reported hoarseness, murmuring, vocal instability, altered jitter and shimmer, voice strangulation at the end of emission, and decreased MPT [12]. Shimmer is lower in people with obesity than that in normal weight individuals [19], and the fundamental frequency is not different between people with obesity and without obesity [10].

One would expect that the changes in the MPT in people with obesity would improve with weight loss, as a consequence of improvement in lung function [20]. Although this hypothesis may be true, such improvement may be slower than expected, not completed at the time of evaluation, or even show interindividual variability. Voice treatment may be indicated to achieve a better voice quality after the surgery even before the expected weight loss.

On the other hand, there are reports showing that there is no change in the MPT in people with obesity after the surgery [16, 17]. In one of these studies, although one-third of patients showed a change in the voice quality after weight loss, it was not detected acoustically, and the MPT did not change [16]. In addition, most of the patients were not class III obese according to the World Health Organization classification [21]. In another study [17], MPT did not change, but the phonation threshold pressure had a trend to decrease with weight loss. These investigations included a small number of individuals, less than ten, but use adequate methodology to evaluate voice. On the other hand, two studies reported an increase in MPT and in fundamental frequency eight months after surgery [11, 15]. These investigations included a larger number of obese patients (more than twenty), but of women only, which differs from our study group.

Altogether, our findings suggest that the MPT improves after weight loss caused by surgical treatment of obesity. The improvement in the voice quality in the postoperative period may be longer than expected, depending more on weight loss than on time. However, we did not find significant influence of weight loss, in percentage of weight loss and in time after surgery, in the direction of longer MPT, which suggested that other factors may have influence of MPT recovery. The results of the influence of weight loss, expressed in percentage, on the MPT almost reach significance with the vowels /A/ and /I/.

This investigation has limitations. MPT was not assessed in the same individual before and after surgical treatment for obesity in most patients and evaluation of MPT in the late postoperative period or after a greater weight loss may have yielded different results. Another limitation was that it was not possible to perform endoscopic evaluation of the larynx before MPT measurement.

In conclusion, MPT is shorter in people with obesity before and after bariatric surgery, with no significant improvement after the treatment. However, MPT showed a tendency to increase in those operated patients.

## Figures and Tables

**Table 1:** Characteristics of people with obesity not submitted to surgical treatment for obesity (group A), individuals submitted to surgical treatment (group B), and healthy controls (group C).

	Controls (*n* = 20)		Obesity no operation (*n* = 52)		Obesity operation (*n* = 62)	
	Mean (SD)	Median	Mean (SD)	Median	Mean (SD)	Median
Age (years)	40.2 (11.9)	42.0	42.6 (11.6)	42.5	41.6 (11.0)	41.0
Weight (kg)	64.3 (7.7)	61.5	143.5 (34.4)	144.7	89.7 (16.4)	87.8
Height (m)	1.7 (0.1)	1.7	1.6 (0.1)	1.6	1.6 (0.1)	1.6
BMI (kg/m^2^)	23.0 (1.8)	23.1	53.2 (11.9)	51.2	33.8 (5.3)	33.6
NC (cm)	33.8 (3.3)	32.5	45.9 (5.4)	44.8	36.9 (3.3)	36.5
FEV_1_ (%)	—	—	85.0 (16.1)	84.5	95.3 (17.0)	95.0
FVC (%)	—	—	87.9 (15.7)	91.2	95.7 (13.7)	96.0
FEV_1_/FVC	—	—	93.7 (11.5)	92.0	91.3 (17.9)	94.5

BMI, body mass index; NC, neck circumference; FEV_1_, forced expiratory volume in one second; FVC, forced vital capacity; FEV_1_/FVC, Tiffeneau index; SD, standard deviation.

**Table 2:** Maximum phonation time (MPT), in seconds, and the S/Z ratio, in people with obesity not submitted to surgical treatment for obesity (group A, *n* = 52), people with obesity submitted to surgical treatment (group B, *n* = 62), and healthy controls (group C, *n* = 20).

	/A/		/I/		/U/		S/Z	
	Mean (SD)	Median	Mean (SD)	Median	Mean (SD)	Median	Mean (SD)	Median
Controls								
Men	16.1 (5.3)	17.1	18.0 (2.6)	18.3	19.0 (2.4)	19.3	0.9 (0.1)	1.0
Women	15.7 (5.1)	14.7	18.0 (5.5)	17.3	17.7 (4.3)	17.0	0.9 (0.2)	0.9
Total	15.7 (5.0)^*∗*^	15.1	18.0 (4.9)^*∗*^	17.3	18.0 (3.6)^*∗*^	18.4	0.9 (0.2)	0.9
Group A								
Men	11.9 (5.3)	12.0	12.4 (5.8)	12.5	12.2 (5.5)	11.2	0.9 (0.2)	1.1
Women	10.3 (3.6)	10.0	11.4 (4.5)	10.5	11.9 (4.7)	11.5	1.0 (0.3)	0.9
Total	10.9 (4.1)	10.8	11.8 (4.9)	11.6	12.0 (5.1)	11.6	1.0 (0.3)	1.0
Group B								
Men	14.0 (6.3)	13.5	14.5 (5.4)	13.7	15.7 (6.0)	15.7	0.9 (0.1)	0.9
Women	11.8 (4.5)	11.6	13.0 (5.8)	12.3	13.2 (5.9)	13.3	0.9 (0.3)	1.0
Total	11.9 (4.8)	11.7	13.0 (5.9)	12.4	13.5 (6.0)	13.7	1.0 (0.2)	1.0

^*∗*^ *p* < 0.05 compared with groups A and B.

**Table 3:** Weight, body mass index (BMI), neck circumference (NC), and maximum phonation time (MPT), in seconds, measured in people with obesity before and after bariatric surgery (Group D, *n* = 15).

	Before surgery		After surgery	
	Mean (SD)	Median	Mean (SD)	Median
Weight (kg)	120.5 (18.9)	117.5	100.6 (15.3)^*∗*^	100
BMI (kg/m^2^)	46.0 (5.5)	46.6	38.4 (4.4)^*∗*^	37.0
NC (cm)	43.4 (3.7)	42.5	39.6 (3.0)^*∗*^	39.0
MPT /a/ (s)	11.8 (4.5)	11.0	13.0 (4.8)^*∗*^	12.1
MPT /I/ (s)	14.1 (5.1)	14.0	13.7 (5.2)	12.6
MPT /U/ (s)	14.2 (5.2)	14.1	13.3 (5.6)	12.7
S/Z	1.0 (0.3)	0.9	0.9 (0.2)	0.9

^*∗*^ *p* < 0.04 compared with preoperative values.

**Table 4:** Spearman correlation coefficient (rho) of maximum phonation time (MPT) and S/Z ratio with weight, height, body mass index (BMI), neck circumference (NC), forced expiratory volume in one second (FEV_1_), forced vital capacity (FVC), and the relation FEV_1_/FVC measured in nonoperated people with obesity (Group A, *n* = 52).

	/A/		/I/		/U/		S/Z	
	rho	*p*	rho	*p*	rho	*p*	rho	*p*
Weight	−0.17	0.06	−0.20	0.02^*∗*^	−0.26	0.01^*∗*^	0.01	0.90
Height	0.23	0.01^*∗*^	0.18	0.04^*∗*^	0.18	0.05^*∗*^	−0.03	0.74
BMI	−0.23	0.01^*∗*^	−0.25	0.01^*∗*^	−0.30	0.01^*∗*^	0.02	0.81
NC	−0.12	0.18	−0.34	0.11	−0.18	0.04^*∗*^	−0.01	0.94
FEV_1_	0.14	0.52	0.07	0.73	0.10	0.63	−0.08	0.71
FVC	0.06	0.77	−0.03	0.88	0.07	0.76	−0.24	0.26
FEV_1_/FVC	0.07	0.75	0.19	0.37	0.05	0.82	0.14	0.51

^*∗*^ *p* < 0.05.

## Data Availability

The individual data used to support the findings of this study are restricted by the Human Research Committee of University Hospital of Ribeirão Preto, Ribeirão Preto Medical School, USP, in order to protect patient privacy. Data are available from the corresponding author via email (rodantas@fmrp.usp.br), for researchers who meet the criteria for access to confidential data.

## References

[1] Racette S. B., Deusinger S. S., Deusinger R. H. (2003). Obesity: overview of prevalence, etiology, and treatment. *Physical Therapy*.

[2] Ortiz V. E., Kwo J. (2015). Obesity: physiologic changes and implications for preoperative management. *BMC Anesthesiology*.

[3] Brazzale D. J., Pretto J. J., Schachter L. M. (2015). Optimizing respiratory function assessments to elucidate the impact of obesity on respiratory health. *Respirology*.

[4] Salome C. M., King G. G., Berend N. (2010). Physiology of obesity and effects on lung function. *Journal of Applied Physiology*.

[5] Steir J., Lunt A., Hart N., Polkey M. I., Moxham J. (2014). Observational study of the effect of obesity on lung volumes. *Torax*.

[6] Teixeira C. A., Santos J. E. d., Silva G. A., Souza E. S. T. d., Martinez J. A. B. (2007). Prevalência de dispnéia e possíveis mecanismos fisiopatológicos envolvidos em indivíduos com obesidade graus 2 e 3. *Jornal Brasileiro de Pneumologia*.

[7] Mafort T. T., Rufino R., Costa C. H., Lopes S. J. (2016). Obesity: systemic and pulmonary complications, biochemical abnormalities, and impairment of lung function. *Multidisciplinary Respiratory Medicine*.

[8] Tagaya M., Nakata S., Yasuma F. (2010). Pathogenetic role of increased nasal resistance in obese patients with obstructive sleep apnea syndrome. *American Journal of Rhinology & Allergy*.

[9] Hora F., Nápolis L. M., Daltro C. (2007). Clinical, anthropometric and upper airway anatomic characteristics of obese patients with obstructive sleep apnea syndrome. *Respiration*.

[10] Celebi S., Yelken K., Develioglu O. N. (2013). Acoustic, perceptual and aerodynamic voice evaluation in an obese population. *The Journal of Laryngology & Otology*.

[11] Souza L. B. R., Pernambuco L. A., Santos M. M., Pereira R. M. (2016). Neck circumference and vocal parameters in women before and after bariatric surgery. *Obesity Surgery*.

[12] Cunha M. G. B., Passerotti G. H., Weber R., Zilberstein B., Cecconello I. (2011). Voice feature characteristic in morbid obese population. *Obesity Surgery*.

[13] Solomon N. P., Garlitz S. J., Milbrath R. L. (2000). Respiratory and laryngeal contributions to maximum phonation duration. *Journal of Voice*.

[14] Speyer R., Bogaardt H. C. A., Passos V. L. (2010). Maximum phonation time: variability and reliability. *Journal of Voice*.

[15] Souza L. B. R., Santos M. M., Pernambuco L. A., Godoy C. M. A., Lima D. M. S. (2018). Effects of weight loss on acoustic parameters after bariatric surgery. *Obesity Surgery*.

[16] Hamdam A. L., Safadi B., Chamseddine G., Kasty M., Turfe Z. A., Ziade G. (2014). Effect of weight loss on voice after bariatric surgery. *Journal of Voice*.

[17] Salomon N. P., Helou L. B., Dietrich-Burns K., Stojadinovic A. (2011). Do obesity and weight loss affect vocal function?. *Seminars in Speech Language*.

[18] Crapo R. O., Morris A. H., Gardner R. M. (1981). Reference spirometric values using techniques and equipment that meet ATS recommendations. *American Review of Respiratory Disease*.

[19] Barsties B., Verfaillie R., Roy N., Maryn Y. (2013). Do body mass index and fat volume influence vocal quality, phonatory range, and aerodynamics in females?. *CoDAS*.

[20] Takahashi T., Ebihara S., Kohzuki M. (2017). Improvement of pulmonary function after comprehensive obesity rehabilitation program in obese patients. *The Tohoku Journal of Experimental Medicine*.

[21] World Health Organization—WHO (1997). *Obesity: Preventing and Managing the Global Epidemic*.

