# Erratum to “Maximum Phonation Time in People with Obesity Not Submitted or Submitted to Bariatric Surgery”

**DOI:** 10.1155/2020/5616713

**Published:** 2020-07-31

**Authors:** Ana Laura Ferreira Fonseca, Wilson Salgado, Roberto Oliveira Dantas

**Affiliations:** ^1^Department of Medicine, Ribeirão Preto Medical School, University of São Paulo, Av. Bandeirantes 3900 Ribeirão Preto SP, São Paulo 14049-900, Brazil; ^2^Department of Surgery and Anatomy, Ribeirão Preto Medical School, University of São Paulo, Av. Bandeirantes 3900 Ribeirão Preto SP, São Paulo 14049-900, Brazil

In the article titled “Maximum Phonation Time in People with Obesity Not Submitted or Submitted to Bariatric Surgery” [1], there was an error in author name, where “Ana Luara Ferreura Fonseca” should be corrected to “Ana Laura Ferreira Fonseca.” The corrected author name is shown in the author list above.
